# Experimental Solution of Chitosan and Nanochitosan on Wettability in Root Dentine: *In Vitro* Model Prior Regenerative Endodontics

**DOI:** 10.1155/2021/8772706

**Published:** 2021-10-31

**Authors:** Fernando Arias Alvarado, Maira Rivero Iriarte, Freddy Jordan Mariño, Sara Quijano-Guauque, León D. Pérez, Yolima Baena, Claudia García-Guerrero

**Affiliations:** ^1^Universidad Nacional de Colombia, Sede Bogotá, Facultad de Odontología, Departamento de Ciencias Básicas y Medicina Oral, Grupo de Investigación INVENDO, Bogotá, Colombia; ^2^Universidad Nacional de Colombia, Sede Bogotá, Facultad de Ciencias, Departamento de Química, Grupo de Investigación Macromoléculas, Bogotá D.C., Colombia; ^3^Universidad Nacional de Colombia, Sede Bogotá, Facultad de Ciencias, Departamento de Farmacia, Grupo de Investigación Sistemas para La Liberación Controlada con Moléculas Biológicamente Activas, Bogotá D.C., Colombia

## Abstract

**Aims:**

To compare the effect of CS and CSnp on the wettability in root dentine with other irrigation protocols with an experimental *in vitro* model prior regenerative endodontics. *Methods and Material*. An *in vitro* experimental study that included eighty hemisected human root distributed into 8 groups: G1- distilled water; G2- 1% NaOCl/17% EDTA; G3- hypochlorous acid 0.025% HOCl, G4- 1% NaOCl/0.025% HOCl/17% EDTA, G5- 0.2 g/100 mL CS, G6- 1% NaOCl/0.2 g/100 mL CS, G7- CSnp, and G8- 1% NaOCl/CSnp. The wettability analysis calculated the contact angle (*θ*) between a drop of a blood-like and root dentinal surface; topographic characterization with scanning electron microscopy (SEM) quantified the diameter and number of tubules per area; spectroscopy infrared analyses (IR-S) identified chemical changes in the inorganic (phosphate/carbonate) and organic phase (amide/methyl). Statistical analysis: a linear mixed model, Kruskal–Wallis, and Holm–Bonferroni correction (*P* < 0.05) were used.

**Results:**

Significantly higher wettability for G2 (27.1 (*P* = 0.0001)) was found. A mean value of 67°±°for experimental groups (*P* = 0.07) was found, and we did not identify differences between them. The SEM identified greater tubular opening and erosion for G4 and greater dentinal permeability per area for NaOCl/CS. IR-S identified dentinal organic integrity with NaOCl-CS/CSnp compared to organic reduction promoted for NaOCl/EDTA.

**Conclusions:**

This *in vitro* dentin determined an indirect association between the wettability and organic contents. The oxidative effect of NaOCl could be neutralized by CS-CSnp, and consequently, the wettability of the substrate decreases.

## 1. Introduction

The process of chemical conditioning the radicular dentin for orthograde or regenerative endodontics comes with tackling challenges such as disinfection, dilution of organic tissue, penetration capacity, and dentin chelation [1]. When root growth stops, chemical conditioning is the primary form of disinfection, and the formation of a suitable microenvironment is conducive to adherence, survival, and differentiation of cellular components, to complete the therapeutic cycle [2].

Despite the irreversible ultrastructural alterations that deproteinate or demineralize the dentin matrix with the use of irrigants [3], sodium hypochlorite (NaOCl) for disinfection and ethylenediaminetetraacetic acid (EDTA) as a chelating agent enhance bioactivity when the blood clot is connected with the inner walls of the root canal [2]. This interfacial integrity between the blood clot and dentin depends on the surface energy when the substrate wettability has been modified [4].

Wettability as physical property is quantified through a contact angle between a liquid and a substrate [5, 6], and it is directly correlated to irrigant type [7], composition [8], roughness, and the tissue topography [9]. Furthermore, it has been demonstrated that surface wettability influences the adhesion and proliferation of different cells [7]. Measurement of wettability values in dentin [8] at the expense of a blood-like solution (BLS) [6] will allow us to recognize the performance of irrigants in regenerative treatment and formulate a hypothesis towards therapeutic fields.

In this perspective, 5.25% NaOCl and 17% EDTA solutions have been found to exhibit hydrophilic interactions with contact angles of 22° and 55°, respectively, thus displaying adequate wettability for disinfection and adhesion of materials or cellular networks [7].

Unfortunately, in regenerative treatment [10], the immature teeth are subjected to the action of NaOCl, which oxidizes and denatures the collagen matrix [3, 8] while EDTA demineralizes [2] and increases dentin erosion in a substrate of less thickness and low mineralization [11, 12]. This fact modifies the physical and chemical dentin properties [13] and reduces the mechanical capacity of the immature teeth [10], despite the success achieved with regenerative treatment. Consequently, recent recommendations suggest decreasing the irrigant concentration or exploring other alternative solutions to tissue preservation.

In the current scenario, research is focused on developing strategies for chemical conditioning that meet the therapeutic objectives and minimize chemically and physically induced damage to the dentinal matrix [14]. Chitosan (CS), a cationic polymer of natural origin, nontoxic, biocompatible, and biodegradable [15] with antimicrobial [16], chelating, and remineralizing properties, [17] has motivated its research as an irrigation alternative in endodontics. With a high molecular weight, CS possesses a lower capacity to penetrate the root dentin when compared to 17% EDTA solution [18], thus justifying the development of CS nanoparticles (CSnp) to enhance its performance [16–18]. With this motivation, the aim was to compare the effect of CS and CSnp on the wettability and chemical structure of surface root dentine with other irrigation protocols with an experimental *in vitro* model prior regenerative endodontics.

## 2. Materials and Methods

An *in vitro* experimental study was carried out under the conditions registered in the ISO11405 standard of 2015, which standardizes the handling of samples for laboratory tests. With the approval of the Ethics Committee of the Faculty of Dentistry (Act 13–17), 40 noncarious, intact, and crack-free human uniradicular teeth were collected and extracted for dental reasons and sectioned longitudinally; they were then distributed randomly in 8 study groups.

### 2.1. Specimen Preparation

The teeth were collected, labeled, and stored in 1% chloramine-T, under refrigeration at 4°C for 1 week, with weekly distilled water (DW) replacements (for a period not exceeding 3 months). The teeth were decoronated at the cemental-enamel junction (CEJ) and hemisected with a low-speed mechanical diamond disc (IsoMet^®^, Buehler Ltd., Lake Bluff, IL, USA) [7]. The integrity of the dentinal tissue of each hemisected root was observed, and the pulp was removed with a K-FlexoFile^®^ 10/.02 (Dentsply Maillefer®). Finally, an ultrasonic wash was performed [6].

### 2.2. Irrigant Synthesis: CS and CSnp

Approximately 200 mg of low-molecular-weight (50–190 kDa) chitosan (Sigma-Aldrich^®^, USA), which is soluble in aqueous acids and has a viscosity of 20–300 cP, was used. This 200 mg CS was mixed with 100.0 mL of a 1% w/v acetic acid solution by magnetic agitation for approximately 2 h.

CSnp was synthesized through the ionic gelation method [19] employing the previously made solution of CS/acetic acid. For making CSnp, a 1 mg/mL solution of sodium tripolyphosphate (Sigma-Aldrich^®^, USA) was added in a dropwise manner to the CS/acetic acid solution and subsequently stirred using a Polytron^®^ homogenizer at 5000 rpm [17].

### 2.3. Sample Distribution and Experimental Groups

The 80 hemiroots were assigned randomly to 8 groups, distributing 10 units per group for a subsequent immersion in in the irrigation solution while ensuring that they were in complete contact with the root dentin, at all times. A volume of 20 mL per solution was used for standardization, including that for refills. The distribution and characterization of the groups (*G*) was performed as follows (*n* = 10): G1- distilled water (5 min) (negative control); G2- 1% NaOCl (ENZOHIP-5^®^; Prodontot^®^ Scientific, Bogota, CO) (two refills of 5 min each) + 17% EDTA (Eufar®, Bogota, CO) (1 min) (positive control); G3- hypochlorous acid 0.025% HOCl (AQUILABS S. A.^®^, Bogota, CO) (two 5-min refills); G4- 1% NaOCl (two refills of 5 min each) + 0.025% HOCl (two refills of 5 min each) + 17% EDTA (1 min); G5- 0.2% CS (3 min); G6- 1% NaOCl (two refills of 5 min each) + 0.2% CS (3 min); G7- CSnp (3 min); and G8- 1% NaOCl (two refills of 5 min each) + CSnp (3 min).

### 2.4. Part 1: *In Vitro* Model for Wettability Analysis

**Figure 1:**
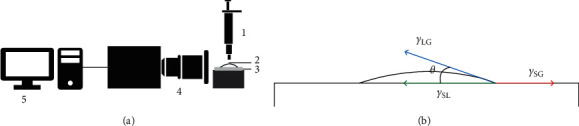
(a) Experimental scheme for the measurement of the contact angle (*θ*). 1- Micropipette, 2- sessile drop (SE), 3- irrigation protocol, 4- camera, and 5- computer. (b) Diagram of the surface tensions: *γ* LG, liquid-gas; *γ* SL, surface-liquid; and *γ* SG, surface-gas.

A BLS of water/glycerin (2:1) with a viscosity of 2-3 cP was prepared, which is close to the value reported for human blood at 37°C [6]. The determination of the static contact angle (*θ*) formed by the treated root dentin surface and the BLS was performed by the sessile drop method [5]. Two markings were made at 3 mm and 9 mm distance from the ACJ to identify the cervical and apical thirds of each hemiroot. Each specimen was placed on a metallic base and fixed with Utility^®^ wax (Hygenic, Coltene^®^, Ohio, USA), and subsequently, 2 *µ*L of the BLS was dispensed vertically with a calibrated micropipette on each of the previously demarcated thirds. A Nikon^®^ D5300 camera with a resolution of 23 megapixels and a Nikon^®^ 18–55 mm lens with a macro photo reversal ring, stabilized with a tripod, captured the instant when the BLS came into contact with the root dentin (Figure 1(a)).

Each photographic image was analyzed by two blind evaluators using ImageJ^®^, version 1.52a (Wayne Rasband, National Institutes of Health, USA, Java 1.8.0_112 (64 bit)). The dropsnake tool was adjusted to the contour of each drop by interpolating B-splines as guide axes for the measurement. To calculate the static contact angle (*θ*), the intersection between the liquid-gas surface tension vector (*γ*_LG_) and the solid-liquid surface tension vector(*γ*_SL_), which corresponds to the dentin surface, was identified (Figure 1(b)). The contact angle for every root third was calculated twice by each observer, and the data were later entered into Microsoft Excel 2007/12 (Microsoft, Redmond, WA, USA) for plotting.

### 2.5. Part 2: Topographical Analysis in Scanning Electron Microscopy

The hemiroots per irrigation group were randomly selected and fixed in holders with carbon adhesive for gold sputtering under vacuum (SPT-20, COXEM BRAND (South Korea, TARGET: Au)). Two-thirds of the cervical and apical roots were selected from each hemiroot. Once the samples were stabilized, they were introduced in the scanning electron microscopy (SEM) equipment (EM-30AX PLUS, COXEM, South Korea) to be observed at 1000×, 2000×, and 5000× magnification.

A qualitative analysis identified the presence of pulp components, dome-shaped calcospherites, areas of visible collagen, shapes of the dentinal tubules, and surface characteristics such as ripples or flattened surfaces [20].

For a quantitative analysis, the images were analyzed at 2000x on an image of size 1,280 *µ*m × 960 *µ*m, using the ImageJ software. The count recorded the number of tubules per field for every third in each hemiroot. Later, two 20 × 20 *µ*m areas were randomly selected from each sample in order to calculate the area of each tubule, with the formula *π∗a∗b* in *µ*m, where *a* is the height and *b* is the width of the tubule [21].

### 2.6. Part 3: Chemical Analysis under Infrared Spectrometry

Three random points on the surface of each sample were identified, and the infrared spectra were obtained on the selected points using a Thermo Scientific Nicolet iS 10 FT-IR spectrometer (Thermo Fisher Scientific, Waltham, MA, USA) equipped with a diamond crystal attenuated total reflectance (ATR) accessory. The spectra were plotted using Origin 8.5 software (OriginLab Corporation, Northampton, MA, USA) [6]. Each recorded spectrum was obtained by averaging the infrared absorption over 10 mm diameter area of the sample with an instrument resolution of 4 cm^−1^ ± 1 cm^−1^. The spectra were recorded in the range of 4000–500 cm^˗1^, corresponding to the vibrations of the inorganic phosphate and carbonate ions or amide bands and methyl groups, present in the dentinal collagen molecule.

### 2.7. Statistical Analysis

For the wettability analysis, a linear model (LM) was implemented to identify inter- and intraobserver correlation. A linear multivariate mixed-effects model (LMM) identified interactions modified by root thirds (cervical and apical) and between the groups. For topographic analysis, a Shapiro–Wilk test identified the distribution of data. Nonparametric tests and Wilcoxon sign rank test (Mann–Whitney U) identified the differences between the root thirds. Kruskal–Wallis and pairwise comparisons using the Wilcoxon rank sum test analyzed the difference between the groups. Finally, a Holm–Bonferroni correction was implemented for multiple hypothesis tests. All data were analyzed through the *R* Sudio software version 1.1.447 (Integrated Development for R, Boston, MA, USA) and Tableau Desktop 2019.3.0 (Salesforce Company, USA) with a confidence interval of 95% (*P* < 0.05).

## 3. Results

### 3.1. Part 1: Wettability Analysis

A total of 80 hemiroots obtained from 54 women and 26 men with a mean age of 28 years were randomly included in eight different experimental groups as an observation unit. According to the observation methodology, a total of 640 angles were obtained. A “substantial” inter- and intraobserver agreement for continuous measurements identified values of 0.96 and 0.98 for the correlation coefficient, respectively (CC: 0.96–0.98; 95% CI (−2.28–2.41) *P* = 0.73) confirming no difference between them [21].

**Figure 2:**
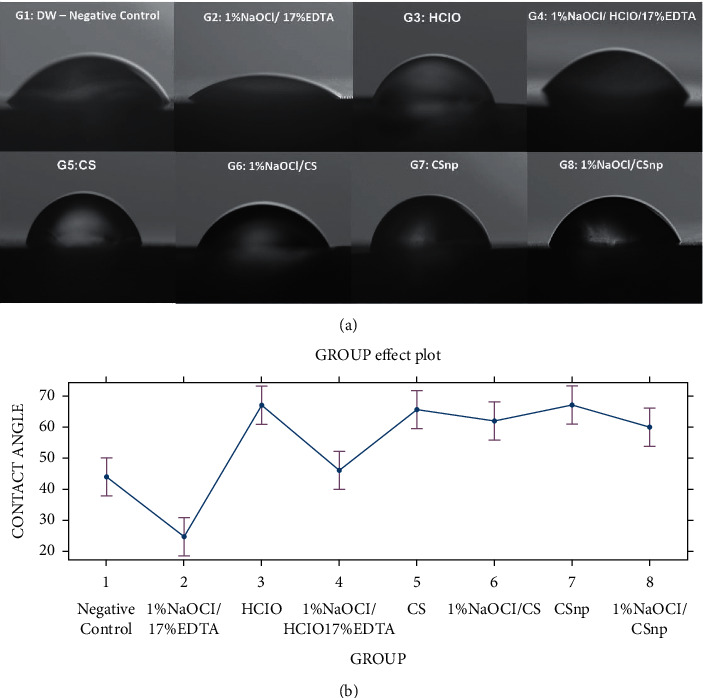
Representative images of wettability. (a) Model *in vitro* with sessile drop on the treated root surface, with each irrigation group as follows: negative control; 1% NaOCl + 17% EDTA; 0.025% HOCl; 1% NaOCl + 0.025% HOCl + 17% EDTA; 0.2% CS; 1% NaOCl + 0.2%CS; CSnp; and 1% NaOCl + CSnp. (b) Statistical lineal model with wettability values for the irrigation group.

Table 1 shows the average contact angle values (*θ*), by group and by root third.  Figure 2(a) includes representative wettability images for each analysis group. The LMM registered a wettability for the group [1% NaOCl + 17% EDTA] comparatively higher than that of the other groups of irrigants, which determined the rejection of Ho (95% CI: −27.33–11.37; *P* = 0.0001). The wettability produced by the four experimental groups with 2% Cs and CSnp alone or with 1% NaOCl was similar without any significant difference between them (95% CI: 0.75–1.63; *P* = 0.07) (Figure 2(b)).

### 3.2. Part 2: Topographical Analysis

**Figure 3:**
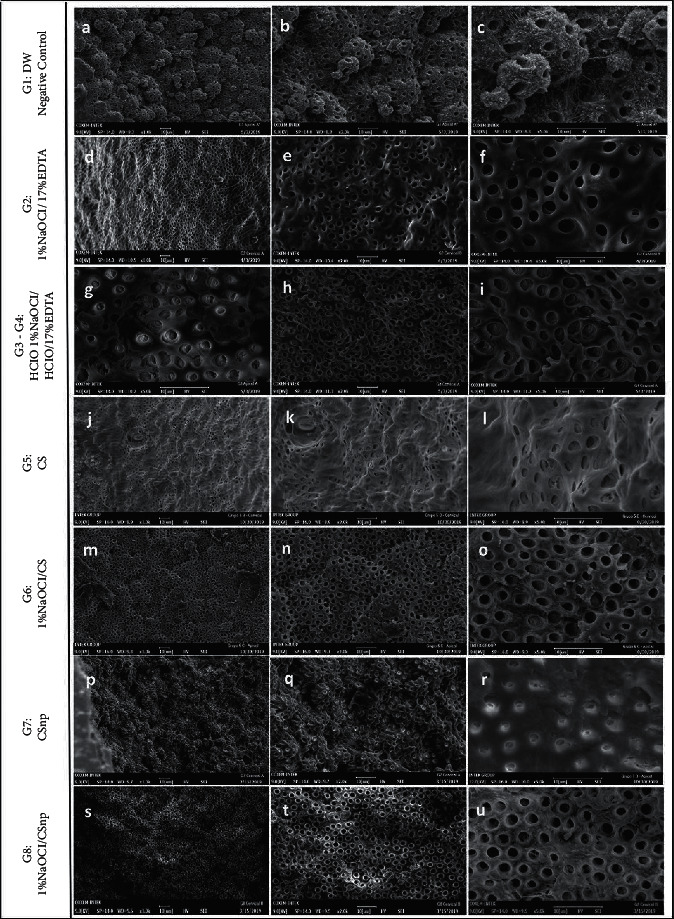
Representative SEM photographs from the cervical and apical thirds, at 1.000×, 2.000×, or 5000×, of each group: visible dentinal tubules were seen in the samples treated with 1% NaOCl+17% EDTA; 1% NaOCl+0.025% HOCl+17% EDTA; 1% NaOCl+0.2%CS; and 1% NaOCl + CSnp. Greater tubular diameter is seen in f, i, and o. Greater number of tubules per field is seen in h, n, and t. Intertubular dentin appearance is seen in l, o, r, and u.

A total of 32 areas were observed. The negative control group (Figure 3, a and b), representing the untreated dentin, showed the presence of calcospherites arranged in the mineral matrix and agglomeration zones with rounded symmetrical tubules with no evidence of peritubular erosion. It was possible to identify a fibrous appearance in the intertubular dentin along with a large number of calcospherites in the apical thirds (Figure 3, c). The [1% NaOCl + 17% EDTA] group shows a clean and flat surface with symmetrical open dentinal tubules and an intertubular zone and no traces of any organic matrix (Figure 3, d–f). The group conditioned with HOCl exclusively identifies an irregular tubular opening with partially occluded dentinal tubules (Figure 3, g). The conjugation between [1% NaOCl + 0.025% HOCl+17% EDTA] presented a large number of exposed tubules and areas of erosion with loss of tubular and intertubular morphology ((Figure 3, h and i). The images corresponding to experimental groups 0.2% CS, [1% NaOCl + 0.2% CS], CSnp, and [1% NaOCl + CSnp] show areas similar to calcospherites, forming superficial wavings that resemble the disposition of intact dentin (Figure 3, j, p, q). 0.2% CS chelation is identified with open dentin tubules with no apparent changes in the intertubular dentin morphology (Figure 3, j–u). In general, groups conditioned with sodium hypochlorite showed a cleaner dentinal surface and homogeneity. The combination of NaOCl and CS or CSnp generated numerous symmetrical tubular openings with no visible areas of erosion (Figure 3, m–o and s–u).

**Figure 4:**
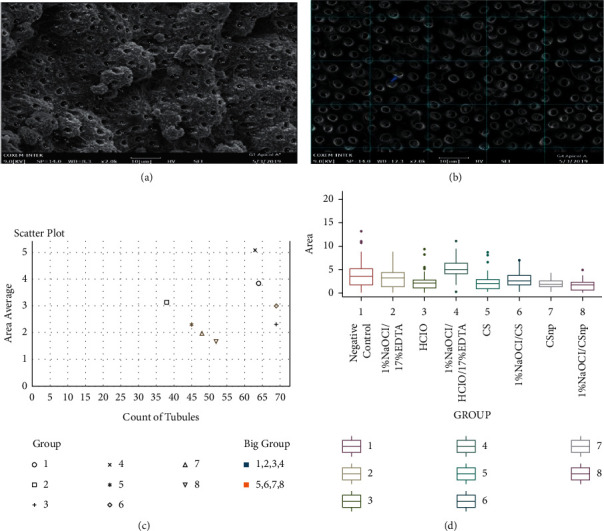
Computational routines for quantitative randomized analysis by SEM x2000. (a) Count of tubules per field. (b) Working field 20 × 20 *µ*m areas; the blue arrow shows the measurement of the tubular diameter. (c) Scatter diagram: area and tubule count per area. (d) Box plot: mean area per tubule.

The range and mean distribution quantified the size for the structures recognized as calcospherites in the range of 5–23 *µ*m (Figure 4(a)). The tubular area was identified by horizontal and vertical diameters varying between 0.3 and 5 *µ*m (Figure 4(b)). A mean area of 3 *µ*m for cervical and 2.9 *µ*m for apical sections indicated no visible difference between the root thirds (*W* = 24,823; *P* value = 0.9575). On the contrary, the chemical conditioning with [1% NaOCl + 0.025% HOCl + 17% EDTA] on mean showed the largest tubular area of 5.1 *µ*m, marking a difference with the other analyzed groups (*K* = 111.61, *P* = 2.2^e ˗ 16^) (Figure 4(c)). Lastly, 0.025% HOCl and [1% NaOCl + 0.2% CS] registered the maximum number of tubules by surface (Figure 4(c)). For the experimental groups with Cs and CSnp alone or combined with 1% NaOCl as the first irrigating, no significant statistical difference was registered in the number of permeable tubules (*P* = 1) (Figure 4(c)). However, including 1% NaOCl within the irrigation protocol with 0.2% CS exhibited a significantly larger diameter (*P* = 0.0006) (Figure 4(d)).

### 3.3. Part 3: Infrared Spectrometry Chemical Analysis

**Figure 5:**
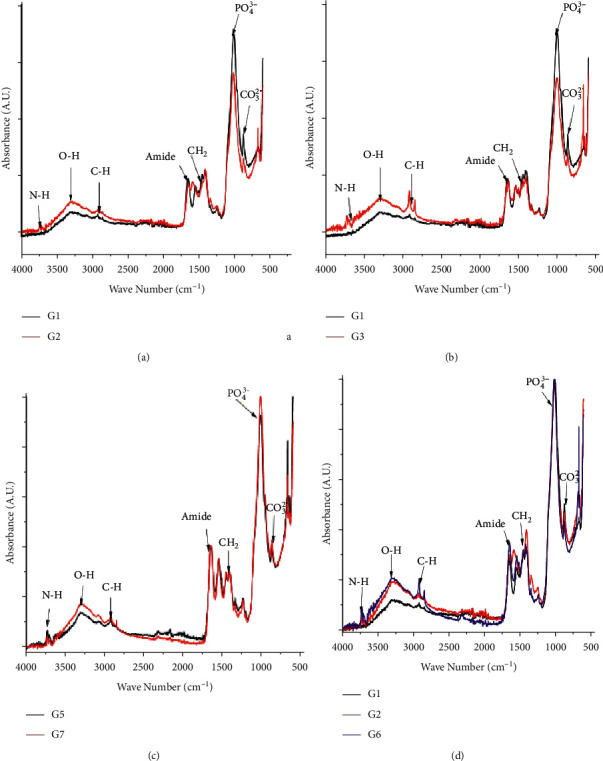
IR representative spectrum. Inorganic phase: phosphate/carbonate ions and the organic phase: amide bands and methyl groups. (a) Negative control and 1% NaOCl+17% EDTA. (b) Negative control and 0.025% HCOl. (c) Experimental groups 0.2% CS and CSnp. (d) Comparative infrared spectrum between negative control [1% NaOCl +17% EDTA] and [1% NaOCl +0.2% CS].

Infrared spectra for [1% NaOCl + 17% EDTA], and [NaOCl + 0.2% CS or CSnp] showed an apparent decrease in the intensity of the peaks corresponding to PO4-3 y CO3-2 (800–1200 cm^˗1^), compared to distilled water as a negative control, confirming the chelating action of EDTA and CS in dispersion or CSnp (Figures 5(a)–5(d)). A lower absorption intensity for the PO4-3 with 0.2% CS than the CSnp establishes better chelating activity for the dispersion 0.2% CS group (Figure 5(c)).

The intensity in absorbance bending (1500 and 1100 cm^−1^) for the organic components (amide and methyl groups) was found to have decreased for [1%NaOCl + 17% EDTA], compared with the negative control, the HOCl, 0.2% CS, and CSnp, confirming the integrity of the organic component of dentin in the absence of NaOCl (Figures 5(a) and 5(b)). Finally, when the NaOCl was combined with CS and CSnp, a higher intensity was observed in the amide and methyl groups, indicating high integrity for the organic component despite the proteolytic effect of hypochlorite (Figures 5(a) and 5(d)).

## 4. Discussion

By extrapolating the experiment to regenerative endodontics using BLS, it was possible to establish that all the irrigation protocols promoting chemical changes, modifying the wettability of the dentinal surface. Additionally, SEM and IR-S analyses confirmed changes in dentin surface topography and its chemical structure, respectively; however, each irrigant imprinted a unique trace on the dentinal surface.

The wettability analysis identified that CS (64.6°) and CSnp (63.2°) generated less surface energy on the dentinal substrate compared to conditioning with the [1% NaOCl + 17% EDTA] solution (27.1°). This difference allowed establishing its relation with the results observed in the topographic and chemical analyses after conditioning. Collagen and hydroxyapatite in dentin act antagonistically on contact with a liquid. The collagen present in the substrate possesses less capacity to break the molecular interactions, thus generating low surface energy. An opposite effect is created by the inorganic portion represented in the apatite crystals [22]. According to this principle, the wettability results depend directly on the modification of the substrate surface by the mechanism of action of each irrigant [5].

Hu et al. [5] observed a significant increase in substrate wettability with 5.25% NaOCl (22.04° ± 3.07), compared to 17% EDTA (54.99° ± 4.06), confirming that as a consequence of NaOCl-generated oxidation, collagen deproteinization generates a hydrophilic and wetted surface [23]. These results coincide with the wettability values obtained in the present work since all groups containing 1% NaOCl (G2, G4, G6, and G8) exhibited a comparatively lower contact angle (Table 1). Notwithstanding, in the present work, the combination of NaOCl and EDTA generated a higher degree of wettability, suggesting that final irrigation with EDTA cannot compensate the organic alteration produced by NaOCl. Then, other alternatives would be more protective for the organic dentin phase. This reflexing is thinking about clinical applicability.

Therefore, if the organic fraction of dentin reduces the free energy on the surface of the substrate [22], then greater oxidation of the collagen molecule will also enhance the wettability of the substrate surface. This premise is complemented by infrared spectrometry results, which show a reduction in the intensity of the bending mode for methyl group and the *C*=O vibrations of the amide groups as part of the dentinal collagen is oxidized in [1% NaOCl + 17% EDTA]. On the contrary, with the presence of nonhydrolyzed collagen fraction for the CS and CSnp group, conditioned with NaOCl in equal concentration and time, corresponding to less oxidative activity on the organic component, by the action of the CS or CSnp, the wettability values identify this change.

Tartari et al. [24] confirmed that the increase in wettability promoted by NaOCl could be associated with a smooth and highly hydrophilic tooth surface. The authors in 2018 identified how the combination of NaOCl + EDTA alters the natural amide III/phosphate ratio between the collagen molecule and the mineralized matrix, the protein elements, demineralizing the inorganic phase, and compromising the organic integrity of dentin [25].

For chelating agents, the wettability of the substrate is related to the surface roughness, which is significantly modified during demineralization [26]. Therefore, high chelation corresponds to a greater irregularity of the tissue surface and, consequently, lower wettability [6]. In our IR spectrometry results, a decrease in the concentration of the inorganic components attributable to the dissolution of phosphate and calcium carbonate crystals from G2 to G8 was noticed, which confirms that EDTA, HOCl and CS, and CSnp possess similar chelation [17]. Likewise, SEM showed a significantly larger tubular diameter for G4 with two chelators, HOCl + EDTA, and a large number of permeable tubules per area were identified. This value was even more remarkable for the experimental groups that supported chelation. The HOCl has an extraordinary bactericidal effect due to its high reduction/oxidation potential (ORP) greater than 1100 mV and has been recommended as an alternative irrigation solution for vital pulp therapy [26]. The inclusion of hypochlorous acid, alone or in combination with EDTA (G4), showed the chelating effect, the integrity of the organic tissue, and the damage that promotes a cumulative chelating effect on the surface topography of the dentin when associated with EDTA.

The results of this research support that CS acts as a chelating agent [27]; however, the difference observed in the degree of wettability concerning the NaOCl + EDTA positive control could be related to a difference in the chelating mechanism. The EDTA, during chelation, releases hydronium ions that increase the acidity of the medium. This phenomenon could contribute to the hydrolysis and/or denaturation of the protein molecules present in dentin [28]. This action could contribute to the hydrolysis and/or denaturation of the protein molecules present in dentin [28]. On the other hand, CS is a weak base, and therefore, its presence generates less impact on the protein material without reducing its chelation capacity and has also been demonstrated in this work. The presence of amino terminal groups (NH2) facilitates metallic complexes that compose the inorganic phase of dentin without increasing the oxidation of collagen previously degraded by NaOCl [26].

Wettability is a physical property that allows the interaction of the dentinal tissue with cellular materials or components [5, 7]. This has fundamental significance in regenerative processes where cellular adhesion to the substrate is required for regeneration to prosper. The construction of a blood-emulated *in vitro* model to analyze the wettability of the dentinal substrate could be the first step to understand the role of chemical conditioning for cellular interaction.

An increase in chelation ability increases cell adhesion [11]. In contrast with a hydrophilic surface, an adequately chelated surface that exposes unbroken collagen and allows the release of bioactive molecules immersed in the matrix would be the most convenient scenario for cell adhesion, viability, and differentiation [29]. For biomedical investigations, hydrophilic surfaces with a contact angle less than 16° and hydrophobic surfaces with a contact angle greater than 102° are not desirable [30]. In the present work, all the wettability values fall within the biologically desirable range. Furthermore, a highly hydrophilic substrate with high surface free energy is postulated as the force necessary for bacteria swimming in the intrabeam space to form the bacterial biofilm [5, 8], thereby suggesting that very high wettability is also undesirable and that intermediate wetting ability values could promote cell adhesion or chemical bonding, without favoring bacterial adhesion. Those events could be considered as future perspectives.

## 5. Conclusions

The use of BLS allowed formulating an *in vitro* model to evaluate the wettability prior to regenerative endodontics. The irrigating solutions modify the dentin wettability, and all wettability values are within the accepted therapeutic range. No indirect association between wettability and the integrity of the organic contents from the dentin was observed; therefore, the highest wettability achieved in the combination NaOCl + EDTA was related to the highest organic oxidation; on the contrary, the implementation of chelating agents such as CS and CSnp demonstrated apparent stability of the organic fraction of root dentin previously treated with NaOCl.

## Figures and Tables

**Table 1:** Mean (*x̄*) and standard deviation (SD) values for the contact angle (*θ*).

Group	Irrigant solution	Coronal third (grades) *X* ± SD	Apical third (grades) *X* ± SD
**G1**	Distilled water	48.9 ± 16.0	44.2 ± 13.7
**G2**	1% NaOCl + 17% EDTA	28.4 ± 8.6	25.9 ± 9.2
**G3**	0.025% HOCl	70.4 ± 11.2	66.7 ± 13.3
**G4**	1% NaOCl + 0.025% HOCl 17% EDTA	47.2 ± 9.1	50.9 ± 8.5
**G5**	0.2% CS	65.7 ± 9.9	69.1 ± 12.0
**G6**	1% NaOCl + 0.2% CS	62.0 ± 4.4	66.3 ± 10.6
**G7**	CSnp	74.4 ± 13.8	62.9 ± 14.8
**G8**	1% NaOCl + CSnp	66.1 ± 9.9	57.5 ± 9.1

## Data Availability

No data were used to support this study.
